# Clinical evaluation of a new fully automated biometer integrating optical low-coherence reflectometry and Placido-disk topography

**DOI:** 10.1186/s40662-026-00492-2

**Published:** 2026-05-31

**Authors:** Yihong Chen, Xinning Yang, Zheng Li, Bowen Tan, Kexin Li, Giacomo Savini, Domenico Schiano-Lomoriello, Xingtao Zhou, Chak Seng Lei, Jinhai Huang

**Affiliations:** 1 Eye Institute and Department of Ophthalmology, Eye & ENT Hospital, Fudan University; NHC Key Laboratory of Myopia and Related Eye Diseases; Key Laboratory of Myopia and Related Eye Diseases, Chinese Academy of Medical Sciences, Shanghai, China; 2 Shanghai Research Center of Ophthalmology and Optometry, Shanghai, China; 3 School of Health Science and Engineering, University of Shanghai for Science and Technology, Shanghai, China; 4 Eye Hospital and School of Ophthalmology and Optometry, Wenzhou Medical University, Wenzhou, Zhejiang China; 5 G.B. Bietti Foundation I.R.C.C.S., Rome, Italy

**Keywords:** Ocular biometry, Optical low coherence reflectometry, Placido-disk topography, Swept-source optical coherence tomography, Repeatability, Agreement

## Abstract

**Background:**

To investigate the precision (intraobserver repeatability and interobserver reproducibility) of the AL550, an automated biometer that integrates optical low-coherence reflectometry with Placido-disk topography, and to assess the inter-device agreement with the IOLMaster 700.

**Methods:**

This prospective study enrolled 112 healthy participants. Each participant underwent three consecutive right-eye measurements by two operators with different levels of experience using the AL550 to assess intraobserver and interobserver variability. Additionally, three measurements were obtained by a skilled operator using the IOLMaster 700 for inter-device comparison. The parameters assessed included axial length (AL), central corneal thickness (CCT), anterior chamber depth (ACD), lens thickness (LT), flattest, steepest, and mean keratometry (Kf, Ks, and Km), astigmatism, and corneal diameter (CD). Measurement precision was analyzed using the within-subject standard deviation (Sw), test–retest repeatability (TRT), coefficient of variation (CoV), and intraclass correlation coefficient (ICC). Inter-device agreement was assessed using paired *t*-tests and Bland–Altman analysis.

**Results:**

The AL550 demonstrated excellent repeatability and reproducibility across all parameters, with low Sw, TRT, and CoV values, and ICCs exceeding 0.939. Inter-device agreement with the IOLMaster 700 was excellent for most parameters, with narrow 95% limits of agreement (LoA) (AL: − 0.09 to 0.08 mm, CCT: − 16.55 to 5.46 μm, ACD: − 0.04 to 0.18 mm, LT: − 0.27 to 0.07 mm, Km: − 0.26 to 0.26 D). However, CD exhibited a wider 95% LoA (− 0.82 to − 0.15 mm).

**Conclusion:**

The AL550 provides highly repeatable and reproducible biometric measurements with minimal operator dependence. Inter-device agreement with the IOLMaster 700 was excellent for all parameters except CD, suggesting that the two devices may be used interchangeably in clinical practice.

## Background

Precise ocular biometry is fundamental to modern ophthalmology, providing critical measurements for both diagnostic evaluation and surgical planning. With the global rise in myopia and the increasing prevalence of its associated vision-threatening complications, accurate axial length (AL) measurement has become essential for monitoring disease progression and guiding therapeutic interventions [1, 2]. In cataract and refractive surgery, reliable biometric measurement is indispensable for intraocular lens (IOL) power calculation and the achievement of target refraction, as even minor measurement errors can result in clinically significant refractive deviations [3, 4].

The advent of optical biometry has largely eliminated the operator dependence associated with the contact ultrasound method [5]. However, measurement variability persists in clinical practice due to factors such as patient cooperation, media opacities, and the constraints of high-volume or resource-limited settings. Ongoing efforts towards greater automation aim to mitigate these sources of variability, thereby enhancing throughput, standardization, and accessibility across diverse clinical environments [6, 7].

The AL550 optical biometer (MediWorks, Shanghai, China) is a new automated device that integrates optical low-coherence reflectometry (OLCR) with Placido-disk topography. The OLCR module captures multiple axial ocular parameters, while the integrated Placido-disk system evaluates anterior corneal curvature and aberrations, facilitating the screening for ectatic disorders [8, 9]. The device incorporates several automation features, including voice-guided instructions, automatic eye tracking and alignment, intelligent capture timing, quality assessment, and dynamic positioning guidance to reduce operator-dependent variability while maintaining measurement accuracy.

Given the absence of prior clinical validation studies for the AL550, rigorous evaluation of its measurement accuracy and reliability is essential before clinical adoption [10]. The present study was therefore designed to comprehensively evaluate the precision of the device. Intraobserver repeatability was examined using both experienced and less experienced operators, while interobserver reproducibility was analyzed to confirm its reliability and automation features. Additionally, measurements obtained with the AL550 were compared with those from the IOLMaster 700 (Carl Zeiss Meditec AG, Jena, Germany), a well-established biometer based on swept-source optical coherence tomography (SS-OCT), to determine inter-device agreement.

## Methods

### Participants

This prospective observational study recruited participants from the Eye and ENT Hospital of Fudan University, Shanghai, China. Each participant underwent a comprehensive ophthalmic assessment, including slit-lamp examination, ophthalmoscopy, and refraction testing. Inclusion criteria were age ≥ 18 years and a best-corrected visual acuity of ≥ 20/20. Contact lens wearers were required to discontinue lens use for at least 2 weeks (soft lenses) or 4 weeks (rigid gas-permeable lenses) prior to measurement. Exclusion criteria comprised the presence of dry eye symptoms, corneal ectasia or scarring, a history of ocular disease, surgery or trauma, and inability to maintain stable fixation. The study protocol was approved by the Institutional Review Board of the Eye and ENT Hospital of Fudan University (Reference No. 2021175) and adhered to the tenets of the Declaration of Helsinki. Written informed consent was obtained from all participants prior to enrolment.

### Instruments

#### AL550

The AL550 is a fully automated biometer that integrates OLCR technology with Placido-disk topography. The OLCR component employs a 1060 nm wavelength light source to measure AL (range, 0.0 to 40.0 mm), central corneal thickness (CCT; range, 200 to 1200 μm), aqueous depth (AQD; range, 0.7 to 8.0 mm), anterior chamber depth (ACD, defined as the sum of CCT and AQD) [11], and lens thickness (LT; range, 1.5 to 6.5 mm). Each OLCR parameter represents the mean of five consecutive measurements. The device converts optical path length to geometrical AL using an equivalent refractive index method; however, the specific conversion algorithm and numerical parameters are proprietary and not disclosed by the manufacturer.

The Placido-disk topographer incorporates a dual-mode lighting system consisting of an 850 nm infrared light-emitting diode (LED) and a 620 nm red light source. It analyzes approximately 125,600 data points to derive keratometric parameters, including flattest, steepest, and mean keratometry (Kf, Ks, and Km), as well as keratometric astigmatism (AST). Measurements are obtained from the central 3.0 mm corneal zone using a standardised keratometric index of 1.3375, within a curvature range of 5.5 to 10.5 mm. The system also provides anterior corneal wavefront aberration data. In addition, an integrated high-definition camera captures en face images of the eye to measure corneal diameter (CD; range, 8.0 to 16.0 mm) and pupillary diameter (PD; range, 1.0 to 13.0 mm).

#### IOLMaster 700

The IOLMaster 700 is a biometer that employs a 1055 nm SS-OCT light source operating at 2000 A-scans per second. The device performs full-length OCT imaging to measure AL (range, 14.0 to 38.0 mm), CCT (range, 200 to 1200 μm), ACD (range, 0.7 to 8.0 mm), and LT (range, 1.0 to 10.0 mm). A telecentric keratometry system measures Kf, Ks, Km, and AST across the 1.5, 2.5, and 3.5 mm central corneal zones (anterior corneal radii range, 5 to 11 mm). For this study, keratometric parameters from the 2.5 mm central zone were recorded using a keratometric index of 1.3375. CD (range, 8 to 16 mm) and PD were measured using the LED light source. According to the manufacturer, AL values are derived using an equivalent refractive index, with additional corrections applied for anatomically non-standard eyes via an undisclosed proprietary algorithm [12].

### Measurement technique

To minimize diurnal variation in corneal parameters, measurements were obtained between 10:00 and 17:00, at least 3 h after awakening [13]. To avoid statistical bias due to inter-eye correlation, only the right eyes were included [10].

All measurements were performed in a controlled, darkened room by two observers with different levels of experience. Observer 1 had more than 3 years of ophthalmic practice and extensive experience in biometric measurements using multiple devices, including the IOLMaster 700. Observer 2 had less than 6 months of ophthalmic experience and limited prior exposure to biometric devices. Neither observer had prior experience with the AL550; both received identical basic training on its operation before the study. The protocol comprised three sets of measurements performed in randomized order: (1) three consecutive measurements with the AL550 by Observer 1; (2) three consecutive measurements with the AL550 by Observer 2; and (3) three consecutive measurements with the IOLMaster 700 by Observer 1.

Participants were seated comfortably with appropriate head stabilisation using chin and forehead rests and were instructed to maintain fixation on the internal target throughout measurement. With the AL550, the operator initiated the automated capture process, during which voice-guided instructions, automatic alignment through eye tracking, and image acquisition were sequentially performed. For the IOLMaster 700, the operator manually aligned the device with the participant’s visual axis and triggered capture. Only measurements meeting each device’s quality criteria (green check mark) were accepted. The following parameters were recorded for analysis: AL, CCT, AQD, ACD, LT, Kf, Ks, Km, AST, and CD.

Corneal astigmatism was analyzed using vector analysis, converting cylinder notation into power vector components, J_0_ and J_45_, calculated as: J_0_ =  − (AST/2) × cos(2α) and J_45_ =  − (AST/2) × sin(2α), with α indicating the astigmatic axis in degrees [14]. The J_0_ vector represents the Jackson cross-cylinder power aligned at 180° and 90°, while J_45_ represents the Jackson cross-cylinder power aligned at 45° and 135°. For visual representation of astigmatic data, double-angle plots were generated, involving doubling the axis angles and plotting them on a polar coordinate system to display the distribution of datasets, the average value (centroid), and the 95% confidence ellipses [15].

### Statistical analysis

Statistical analyses were performed using MedCalc (version 19.8, MedCalc Software Ltd., Ostend, Belgium), SPSS (version 25.0, IBM Corporation, New York, USA), and Microsoft Excel (version 2024, Microsoft Corp., Washington, USA). Normality of data distribution was assessed using the Kolmogorov–Smirnov test (*P* > 0.05). Continuous variables are reported as mean ± standard deviation (SD). Statistical significance was set at *P* < 0.05.

The precision of the AL550 was assessed using multiple statistical indices. The within-subject standard deviation (Sw) was computed as the square root of the within-subject variance to quantify measurement variability. Test–retest repeatability (TRT) was defined as 2.77 times Sw, representing the 95% measurement error interval [16]. The coefficient of variation (CoV) was calculated as Sw divided by the overall mean and expressed as a percentage. For parameters with mean values close to zero (AST, J_0_, and J_45_), CoV was not calculated, as it would lack meaningful interpretation. The intraclass correlation coefficient (ICC), derived using a two-way mixed-effects model with absolute agreement, was used to assess measurement consistency; values approaching 1.0 indicate excellent reliability [17].

Agreement between the AL550 and IOLMaster 700 was evaluated using paired *t*-tests and Bland–Altman analysis. The 95% limits of agreement (LoA) were defined as the mean difference ± 1.96 SD [18].

Sample size determination followed statistical recommendations for precision and agreement studies provided by McAlinden et al. For a study design involving three repeated measurements, at least 96 participants are required to estimate precision within a 10% confidence level [10]. For clinical agreement studies, a minimum sample size of 100 subjects is recommended [16].

## Results

A total of 112 right eyes from 112 healthy participants (59 males and 53 females) were included in the analysis. The mean age was 26.45 ± 6.58 years (range, 18 to 43 years). The mean spherical equivalent refractive error was − 5.25 ± 2.07 dioptres (D) (range, − 12.00 to − 1.00 D), with a mean spherical power of − 4.75 ± 2.02 D (range, − 11.50 to − 0.25 D), and a mean cylindrical power of − 1.01 ± 0.72 D (range, − 3.75 to 0.00 D).

**Fig. 1:**
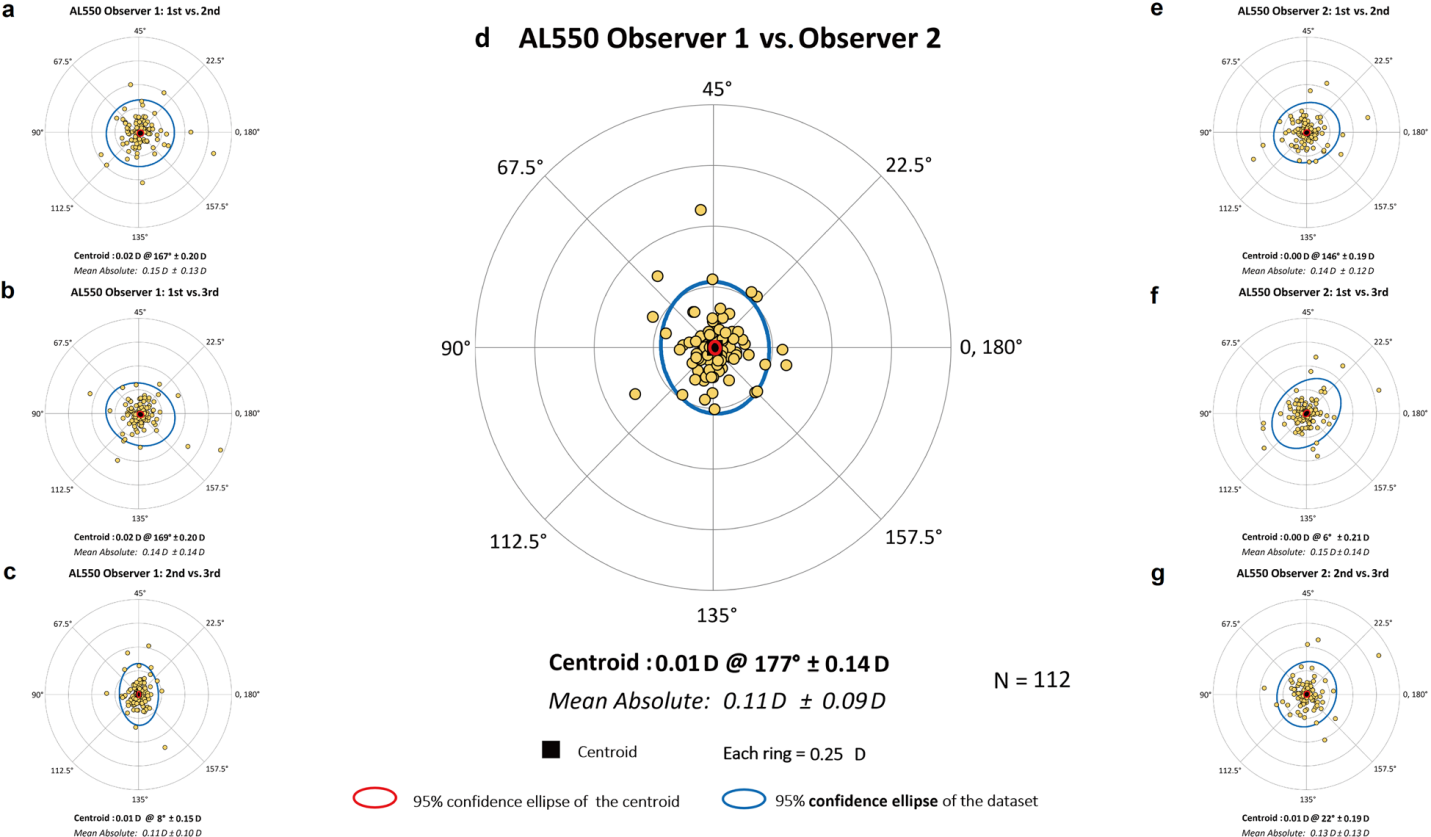
Double-angle plots illustrating intraobserver and interobserver differences in corneal astigmatism measurements. **a**–**c** Show intraobserver repeatability for Observer 1; **d** shows interobserver reproducibility between Observers 1 and 2; and **e**–**g** show intraobserver repeatability for Observer 2

Table 1 presents intraobserver repeatability metrics (Sw, TRT, CoV, and ICC). The AL550 device demonstrated excellent repeatability across all parameters, with low Sw and TRT values for both observers. The CoV values remained consistently low (0.07%–1.12%), with ICCs exceeding 0.939 for all parameters. The interobserver reproducibility analysis (Table 2) also showed excellent agreement between the experienced and junior operators. Sw and TRT values remained at very low levels, while both CoV values (0.04%–0.68%) and ICCs (> 0.962) further confirmed high consistency between operators with different levels of experience. Intraobserver and interobserver astigmatic differences (Fig. 1) were predominantly within 0.25 D, with all centroids tightly clustered around 0.00 D.

**Table 1:** Intraobserver repeatability analysis of the AL550

Parameter	Observer	Mean ± SD	Sw	TRT	CoV (%)	ICC (95% CI)
AL (mm)	1^st^	25.78 ± 1.23	0.02	0.05	0.07	1.000 (1.000 to 1.000)
	2^nd^	25.78 ± 1.24	0.02	0.05	0.07	1.000 (1.000 to 1.000)
CCT (μm)	1^st^	547.03 ± 29.50	2.81	7.79	0.51	0.991 (0.988 to 0.994)
	2^nd^	546.72 ± 29.32	2.82	7.82	0.52	0.991 (0.987 to 0.993)
AQD (mm)	1^st^	3.19 ± 0.26	0.03	0.07	0.84	0.989 (0.985 to 0.992)
	2^nd^	3.19 ± 0.25	0.03	0.07	0.80	0.990 (0.986 to 0.993)
ACD (mm)	1^st^	3.74 ± 0.25	0.03	0.07	0.71	0.989 (0.985 to 0.992)
	2^nd^	3.74 ± 0.25	0.03	0.07	0.67	0.990 (0.987 to 0.993)
LT (mm)	1^st^	3.53 ± 0.29	0.04	0.11	1.12	0.982 (0.975 to 0.987)
	2^nd^	3.53 ± 0.29	0.04	0.10	1.03	0.985 (0.979 to 0.989)
Kf (D)	1^st^	42.47 ± 1.54	0.14	0.38	0.32	0.992 (0.989 to 0.994)
	2^nd^	42.46 ± 1.54	0.14	0.39	0.33	0.992 (0.989 to 0.994)
Ks (D)	1^st^	43.79 ± 1.64	0.13	0.37	0.31	0.993 (0.991 to 0.995)
	2^nd^	43.78 ± 1.65	0.15	0.41	0.34	0.992 (0.989 to 0.994)
Km (D)	1^st^	43.13 ± 1.56	0.13	0.35	0.30	0.993 (0.991 to 0.995)
	2^nd^	43.12 ± 1.56	0.14	0.38	0.32	0.992 (0.989 to 0.994)
AST (D)	1^st^	1.32 ± 0.68	0.09	0.24	–	0.984 (0.979 to 0.989)
	2^nd^	1.32 ± 0.69	0.09	0.25	–	0.983 (0.977 to 0.988)
J_0_ (D)	1^st^	− 0.60 ± 0.38	0.05	0.13	–	0.985 (0.980 to 0.989)
	2^nd^	− 0.61 ± 0.39	0.05	0.14	–	0.984 (0.978 to 0.988)
J_45_ (D)	1^st^	− 0.02 ± 0.20	0.05	0.13	–	0.944 (0.925 to 0.960)
	2^nd^	− 0.02 ± 0.19	0.05	0.13	–	0.939 (0.918 to 0.956)
CD (mm)	1^st^	11.48 ± 0.42	0.10	0.27	0.84	0.949 (0.931 to 0.963)
	2^nd^	11.47 ± 0.42	0.10	0.27	0.86	0.947 (0.928 to 0.961)

*ACD* = anterior chamber depth; *AL* = axial length; *AQD* = anterior aqueous depth; *AST* = astigmatism magnitude; *CCT* = central corneal thickness; *CD* = corneal diameter; *CI* = confidence interval; *CoV* = within-subject coefficient of variation; *ICC* = intraclass correlation coefficient; *J*_*0*_ = Jackson cross-cylinder at 0°; *J*_*45*_ = Jackson cross-cylinder at 45°; *Kf* = flattest keratometry; *Km* = mean keratometry; *Ks* = steepest keratometry; *LT* = lens thickness; *SD* = standard deviation; *Sw* = within-subject standard deviation; *TRT* = test–retest repeatability (2.77 Sw)

**Table 2:** Interobserver reproducibility analysis of the AL550

Parameter	Mean ± SD	S_w_	TRT	CoV (%)	ICC (95% CI)
AL (mm)	25.78 ± 1.23	0.01	0.03	0.04	1.000 (1.000 to 1.000)
CCT (μm)	546.88 ± 29.35	1.98	5.49	0.36	0.995 (0.993 to 0.997)
AQD (mm)	3.19 ± 0.25	0.02	0.05	0.57	0.995 (0.993 to 0.997)
ACD (mm)	3.74 ± 0.25	0.02	0.05	0.48	0.995 (0.993 to 0.997)
LT (mm)	3.53 ± 0.29	0.02	0.07	0.68	0.993 (0.990 to 0.995)
Kf (D)	42.46 ± 1.54	0.08	0.23	0.19	0.997 (0.996 to 0.998)
Ks (D)	43.78 ± 1.64	0.09	0.25	0.21	0.997 (0.995 to 0.998)
Km (D)	43.13 ± 1.55	0.08	0.22	0.19	0.997 (0.996 to 0.998)
AST (D)	1.32 ± 0.68	0.06	0.17	–	0.992 (0.988 to 0.994)
J_0_ (D)	− 0.61 ± 0.38	0.03	0.09	–	0.993 (0.991 to 0.996)
J_45_ (D)	− 0.02 ± 0.19	0.04	0.10	–	0.962 (0.946 to 0.974)
CD (mm)	11.48 ± 0.42	0.06	0.17	0.52	0.979 (0.970 to 0.986)

*ACD* = anterior chamber depth; *AL* = axial length; *AQD* = anterior aqueous depth; *AST* = astigmatism magnitude; *CCT* = central corneal thickness; *CD* = corneal diameter; *CI* = confidence interval; *CoV* = within-subject coefficient of variation; *ICC* = intraclass correlation coefficient; *J*_*0*_ = Jackson cross-cylinder at 0°; *J*_*45*_ = Jackson cross-cylinder at 45°; *Kf* = flattest keratometry; *Km* = mean keratometry; *Ks* = steepest keratometry; *LT* = lens thickness; *SD* = standard deviation; *Sw* = within-subject standard deviation; *TRT* = test–retest repeatability (2.77 Sw)

**Fig. 2:**
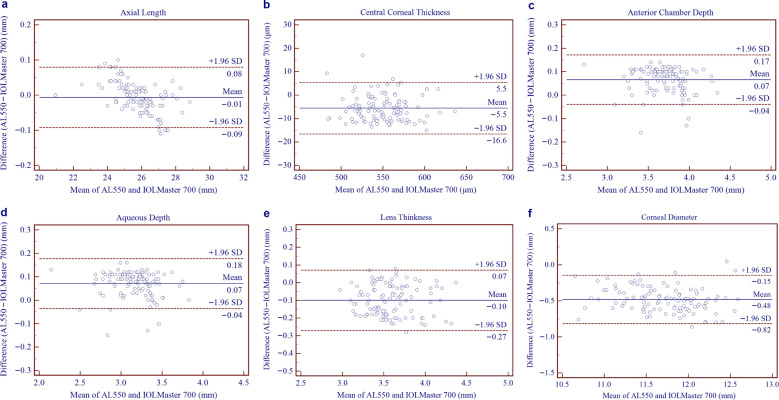
Bland–Altman plots demonstrating the agreement between AL550 and IOLMaster 700. **a** Axial length; **b** Central corneal thickness; **c** Anterior aqueous depth; **d** Anterior chamber depth; **e** Lens thickness; **f** Corneal diameter. The continuous blue line represents the mean difference. The upper and lower red dotted lines represent the 95% limits of agreement

**Fig. 3:**
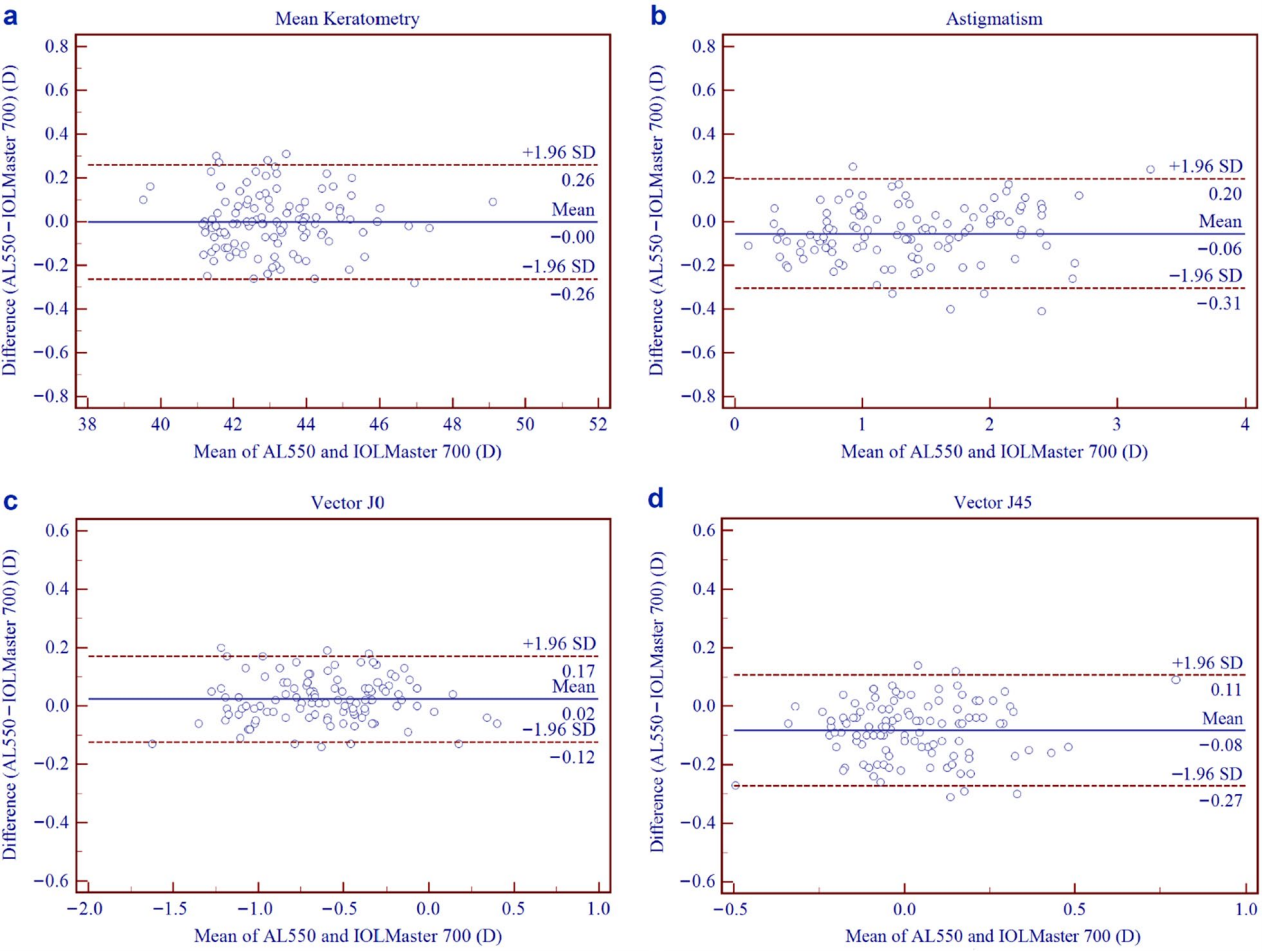
Bland–Altman plots demonstrating the agreement between AL550 and IOLMaster 700. **a** Mean keratometry; **b** Corneal astigmatism; **c** J_0_ vector; **d** J_45_ vector. The continuous blue line represents the mean difference. The upper and lower red dotted lines represent the 95% limits of agreement

**Fig. 4:**
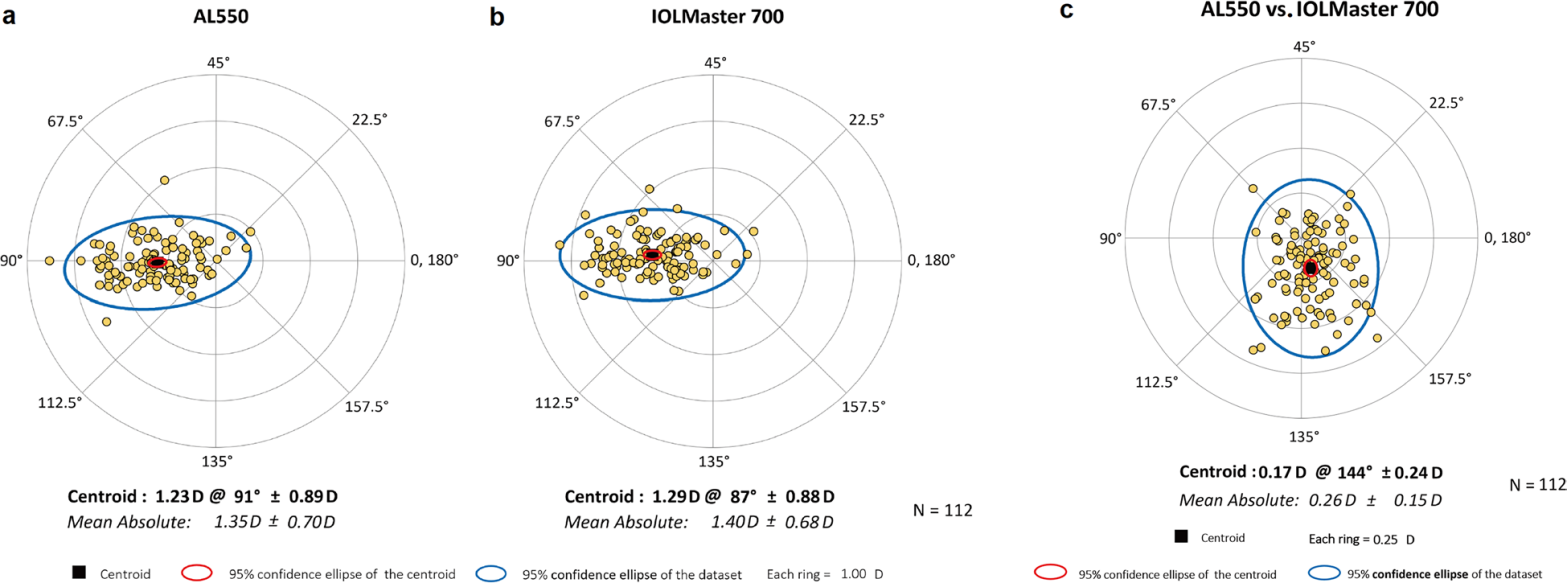
Double-angle plots illustrating corneal astigmatism measurements. **a** AL550; **b** IOLMaster 700; **c** The inter-device differences

Table 3 presents the biometric parameters measured using the AL550 and IOLMaster 700 devices. Table 4 summarises the inter-device comparisons, including corresponding *P* values and 95% LoAs derived from Bland–Altman analyses (Figs. 2, 3). No statistically significant differences were observed for AL, Kf, Ks, and Km (all *P* > 0.05). In contrast, CCT, AQD, ACD, AST, J_0_, and J_45_ showed statistically significant differences (all *P* < 0.05). However, these differences were of limited clinical relevance given the narrow 95% LoA ranges. In contrast, CD demonstrated a larger mean difference (− 0.48 ± 0.17 mm) and wider LoA (− 0.82 to − 0.15 mm). Vector analysis using double-angle plots (Fig. 4) revealed comparable astigmatic distributions between devices. The AL550 measured a mean AST of 1.23 D at 91° ± 0.89 D, while the IOLMaster 700 measured 1.29 D at 87° ± 0.88 D. Inter-device astigmatic differences were within clinically acceptable limits, with a difference vector centroid of 0.17 D at 144° ± 0.24 D, indicating minimal systematic bias between the instruments.

**Table 3:** Biometric parameters measured by the AL550 and IOLMaster 700

Parameter	AL550			IOLMaster 700		
	Mean ± SD	Minimum	Maximum	Mean ± SD	Minimum	Maximum
AL (μm)	25.78 ± 1.23	20.94	28.82	25.79 ± 1.26	20.94	28.84
CCT (μm)	547.03 ± 29.50	479.07	632.70	552.58 ± 29.65	478.33	639.67
AQD (mm)	3.74 ± 0.25	2.78	4.35	3.67 ± 0.26	2.65	4.34
ACD (mm)	3.19 ± 0.26	2.21	3.83	3.12 ± 0.26	2.08	3.83
LT (mm)	3.53 ± 0.29	2.95	4.37	3.63 ± 0.29	2.99	4.43
Kf (D)	42.47 ± 1.54	39.00	48.10	42.44 ± 1.54	38.86	48.03
Ks (D)	43.79 ± 1.64	39.90	50.20	43.82 ± 1.65	39.85	50.10
Km (D)	43.13 ± 1.56	39.58	49.15	43.13 ± 1.56	39.48	49.06
AST (D)	1.32 ± 0.68	0.05	3.38	1.38 ± 0.66	0.16	3.14
J_0_ (D)	− 0.60 ± 0.38	− 1.69	0.37	− 0.63 ± 0.38	− 1.56	0.43
J_45_ (D)	− 0.02 ± 0.20	− 0.63	0.84	0.06 ± 0.19	− 0.36	0.75
CD (mm)	11.48 ± 0.42	10.31	12.52	11.96 ± 0.46	10.73	12.87

*ACD* = anterior chamber depth; *AL* = axial length; *AQD* = anterior aqueous depth; *AST* = astigmatism magnitude; *CCT* = central corneal thickness; *CD* = corneal diameter; *J*_*0*_ = Jackson cross-cylinder at 0°; *J*_*45*_ = Jackson cross-cylinder at 45°; *Kf* = flattest keratometry; *Km* = mean keratometry; *Ks* = steepest keratometry; *LT* = lens thickness; *SD* = standard deviation

**Table 4:** Inter-device agreement analysis between the AL550 and IOLMaster 700

Parameter	Mean difference ± SD	*P* value	95% LoA
AL (μm)	− 0.01 ± 0.04	0.109	− 0.09 to 0.08
CCT (μm)	− 5.55 ± 5.61	< 0.001	− 16.55 to 5.46
AQD (mm)	0.07 ± 0.05	< 0.001	− 0.04 to 0.17
ACD (mm)	0.07 ± 0.05	< 0.001	− 0.04 to 0.18
LT (mm)	− 0.10 ± 0.09	< 0.001	− 0.27 to 0.07
Kf (D)	0.03 ± 0.14	0.073	− 0.24 to 0.29
Ks (D)	− 0.03 ± 0.16	0.054	− 0.34 to 0.28
Km (D)	0.00 ± 0.13	0.882	− 0.26 to 0.26
AST (D)	− 0.06 ± 0.13	< 0.001	− 0.31 to 0.20
J_0_ (D)	0.02 ± 0.08	0.001	− 0.12 to 0.17
J_45_ (D)	− 0.08 ± 0.10	< 0.001	− 0.27 to 0.11
CD (mm)	− 0.48 ± 0.17	< 0.001	− 0.82 to − 0.15

*ACD* = anterior chamber depth; *AL* = axial length; *AQD* = anterior aqueous depth; *AST* = astigmatism magnitude; *CCT* = central corneal thickness; *CD* = corneal diameter; *J*_*0*_ = Jackson cross-cylinder at 0°; *J*_*45*_ = Jackson cross-cylinder at 45°; *Kf* = flattest keratometry; *Km* = mean keratometry; *Ks* = steepest keratometry; *LT* = lens thickness; *LoA* = limits of agreement; *SD* = standard deviation

Significant proportional bias was observed for AL (slope =  − 0.019, 95% confidence interval [CI]: − 0.025 to − 0.014, *P* < 0.001) and CD (slope =  − 0.091, 95% CI: − 0.162 to − 0.019, *P* = 0.014). No significant proportional bias was detected for CCT, ACD, AQD, LT, keratometric parameters (Kf, Ks, Km), AST magnitude, or vector components (J_0_ and J_45_) (all *P* > 0.05).

## Discussion

This study provides the first reported clinical validation of the AL550, a novel automated biometer that combines OLCR technology with Placido-disk topography. Prior to implementing any new device in clinical practice, its measurement precision and agreement with established reference devices must be verified. The present findings demonstrate that the AL550 exhibits excellent precision for AL and anterior segment measurements, and maintains its reliability even when used by operators with limited experience. Furthermore, excellent inter-device agreement was observed with the IOLMaster 700, a widely used SS-OCT biometer, across most ocular parameters.

The repeatability and reproducibility achieved by the AL550 are comparable to those previously reported for other modern biometers, including the IOLMaster 700, Anterion (Heidelberg Engineering, Heidelberg, Germany), OA-2000 (Tomey, Nagoya, Japan), Eyestar 900 and Lenstar LS 900 (Haag-Streit AG, Koeniz, Switzerland), all of which demonstrate high precision, with Sw values of < 0.03 mm for AL, < 0.20 D for keratometry and < 4.00 μm for CCT [19–22]. Most significantly, interobserver reproducibility between operators with differing levels of experience showed minimal variability, with CoV values ranging from 0.04% to 0.68% and ICCs from 0.962 to 1.000.

Several previous studies have reported that SS-OCT-based devices may exhibit slightly superior repeatability compared with OLCR-based devices [19, 22, 23], potentially due to higher scanning speed and axial resolution. In the present study, however, the AL550 achieved repeatability metrics comparable to those reported for SS-OCT devices, with low Sw, TRT, and CoV values, and high ICCs. These findings indicate that the modern OLCR technology, when combined with advanced automation features, can achieve precision levels comparable to SS-OCT devices for most clinical applications. The clinical significance of any marginal differences in repeatability between technologies is likely minimal, as both approaches provide measurements well within clinically acceptable ranges.

The AL550 demonstrated clinically acceptable agreement with the IOLMaster 700 across most parameters. The negative proportional bias for AL (slope =  − 0.019) indicated that the AL550 tended to yield slightly higher AL values in shorter eyes and slightly lower AL values in longer eyes compared with the IOLMaster 700. However, the mean AL difference of − 0.01 mm was not statistically significant (*P* = 0.109), and the 95% LoA range (− 0.09 to 0.08 mm) remained within clinically acceptable limits, given that a 0.1 mm variation in AL corresponds to approximately 0.27 D of IOL power [3]. Martínez-Plaza et al. [24] reported comparable findings when evaluating two new optical biometers, the MYAH (Topcon, Tokyo, Japan) and the Myopia Master (Oculus, Wetzlar, Germany), both of which showed interchangeable AL measurements with 95% LoA ranging from − 0.09 to 0.15 mm. Similarly, Sabur and Takes [25] found good agreement between the IOLMaster 700 and MYAH, reporting a narrow 95% LoA (− 0.11 to 0.06 mm).

Anterior segment parameters, including CCT, ACD, and LT, are essential for various clinical applications, such as IOL selection, refractive surgery planning, and glaucoma assessment [26–28]. Arriola-Villalobos et al. [29] reported excellent agreement between the Lenstar LS 900 and IOLMaster 700, with a narrow 95% LoA for CCT (− 9.03 to 14.05 μm), ACD (− 0.09 to 0.06 mm), and LT (− 0.16 to 0.23 mm). Similarly, Domínguez-Vicent et al. [22] reported that the fully automated SS-OCT-based Eyestar 900 and the OLCR-based LS 900 could be used interchangeably, with 95% LoA of − 4.77 to 11.01 μm for CCT, − 0.09 to 0.12 mm for ACD, and − 0.20 to 0.15 mm for LT. In the present study, the AL550 demonstrated comparable agreement with the IOLMaster 700, with 95% LoA of − 16.55 to 5.46 μm for CCT, − 0.04 to 0.18 mm for ACD, and − 0.27 to 0.07 mm for LT. The observed systematic differences between devices may arise from several factors. First, technological differences between OLCR and SS-OCT may influence tissue penetration and reflectivity, as each technology employs distinct signal-processing approaches to identify anatomical boundaries. Second, variations in tissue boundary detection algorithms across manufacturers can result in differences in interface delineation. Third, device-specific calibration processes may contribute to small measurement discrepancies. While these biases are statistically significant, their clinical relevance remains limited. Given that a 0.1 mm difference in either ACD or LT could lead to approximately 0.1 D of IOL power change [3], the maximum absolute LoA observed would result in less than 0.25 D of IOL power deviation, indicating that these minimal differences could be considered clinically acceptable.

For keratometric measurements, no statistically significant differences were observed, with narrow 95% LoA: Kf (− 0.24 to 0.29 D; *P* = 0.073), Ks (− 0.34 to 0.28 D; *P* = 0.054), and Km (− 0.26 to 0.26 D; *P* = 0.882). These findings are consistent with previous inter-device comparisons between OLCR and SS-OCT technologies. Hoffer et al. [30], reported 95% LoA ranging from − 0.37 to 0.43 D for Kf, − 0.52 to 0.53 D for Ks, and − 0.31 to 0.34 D for Km, with no statistically significant differences. Likewise, Domínguez-Vicent et al. [22] reported 95% LoA of − 0.21 to 0.19 D for Kf and − 0.30 to 0.25 D for Ks when comparing OLCR- and SS-OCT-based devices, also without significant differences. Furthermore, vector analysis and double-angle plots showed that most astigmatic differences were within 0.50 D, indicating excellent inter-device agreement.

Regarding CD measurements, the AL550 consistently produced lower values than the IOLMaster 700, with a mean difference of − 0.48 mm (*P* < 0.001) and 95% LoA of − 0.82 to − 0.15 mm. As CD is commonly used to estimate phakic IOL size for refractive surgery, and such lenses are manufactured in approximately 0.5 mm increments, this discrepancy is clinically relevant. The relatively wide 95% LoA indicates substantial inter-device variability, precluding interchangeable use for CD assessment. This inconsistency likely reflects differences in measurement algorithms or edge-detection methods among manufacturers. Supporting this, a review by Muzyka-Woźniak et al. [31], which included 41 studies comparing 19 devices, concluded that nearly all analysed studies demonstrated a lack of interchangeability for CD measurements.

A key limitation of this study is the lack of access to detailed technical specifications regarding the conversion of optical path length to geometrical AL in both devices. Although both the AL550 and IOLMaster 700 employ equivalent refractive index methods for this conversion, the specific conversion algorithms, numerical values of the equivalent refractive indices, and any proprietary correction factors applied by each manufacturer remain undisclosed [12]. This limited our ability to elucidate the underlying mechanisms of the observed systematic differences and proportional bias.

Future research should further explore how the device performs under more challenging conditions, such as dense cataracts, corneal pathology, or other factors that may compromise measurement accuracy. Its capability to characterise corneal irregularities using Placido-disk topography also warrants evaluation for potential application in corneal disease screening. Furthermore, comparisons with other established biometers may provide additional insight into measurement consistency across different platforms.

## Conclusion

In summary, the AL550, a recently developed fully automated OLCR-based biometer, demonstrated excellent intraobserver repeatability and interobserver reproducibility. Its automation features standardise the measurement process and minimise operator dependence. Compared with the SS-OCT-based biometer IOLMaster 700, the narrow 95% LoA indicates high inter-device agreement across all biometric parameters except CD, supporting their interchangeable use for most measurements.

## Data Availability

All data analyzed in this study are included in the published article.

## Abbreviations

OLCR: Optical low-coherence reflectometry
SS-OCT: Swept-source optical coherence tomography
AL: Axial length
CCT: Central corneal thickness
AQD: Aqueous depth
ACD: Anterior chamber depth
LT: Lens thickness
Kf: Flattest meridian keratometry
Ks: Steepest meridian keratometry
Km: Mean keratometry
AST: Astigmatism
CD: Corneal diameter
Sw: Within-subject standard deviation
TRT: Test–retest repeatability
CoV: Coefficient of variation
ICC: Intraclass correlation coefficient
