# Publisher Correction: Clinical evaluation of a new fully automated biometer integrating optical low-coherence reflectometry and Placido-disk topography

**DOI:** 10.1186/s40662-026-00500-5

**Published:** 2026-07-07

**Authors:** Yihong Chen, Xinning Yang, Zheng Li, Bowen Tan, Kexin Li, Giacomo Savini, Domenico Schiano-Lomoriello, Xingtao Zhou, Chak Seng Lei, Jinhai Huang

**Affiliations:** 1 Eye Institute and Department of Ophthalmology, Eye & ENT Hospital, Fudan University; NHC Key Laboratory of Myopia and Related Eye Diseases; Key Laboratory of Myopia and Related Eye Diseases, Chinese Academy of Medical Sciences, Shanghai, China; 2 Shanghai Research Center of Ophthalmology and Optometry, Shanghai, China; 3 School of Health Science and Engineering, University of Shanghai for Science and Technology, Shanghai, China; 4 Eye Hospital and School of Ophthalmology and Optometry, Wenzhou Medical University, Wenzhou, Zhejiang China; 5 G.B. Bietti Foundation I.R.C.C.S., Rome, Italy

**Publisher Correction: Eye and Vision (2026) 13:22** 10.1186/s40662-026-00492-2

After publication of the article, it was brought to our attention that the corresponding author identifier was not added for the co-corresponding author Jinhai Huang. In the accepted manuscript, Jinhai Huang was listed as a co-corresponding author. However, this designation was inadvertently omitted during the production process.

The original publication has been updated. The publisher apologizes to the authors and readers for the inconvenience caused.

